# Drug-Induced Nephrotoxicity Assessment in 3D Cellular Models

**DOI:** 10.3390/mi13010003

**Published:** 2021-12-21

**Authors:** Pengfei Yu, Zhongping Duan, Shuang Liu, Ivan Pachon, Jianxing Ma, George P. Hemstreet, Yuanyuan Zhang

**Affiliations:** 1 Difficult & Complicated Liver Diseases and Artificial Liver Center, Fourth Department of Liver Disease, Beijing Youan Hospital, Capital Medical University, Beijing 100069, China; eddie.yu@mail.ccmu.edu.cn (P.Y.); duan@ccmu.edu.cn (Z.D.); shuangliu186@163.com (S.L.); 2 Beijing Municipal Key Laboratory of Liver Failure and Artificial Liver Treatment Research, Beijing Youan Hospital, Capital Medical University, Beijing 100069, China; 3 Wake Forest Institute for Regenerative Medicine, Wake Forest University Health Sciences, Winston-Salem, NC 27157, USA; pachig18@wfu.edu; 4 Department of Biochemistry, Wake Forest University Health Sciences, Winston-Salem, NC 27157, USA; jian-xing-ma@ouhsc.edu; 5 Omaha Veterans Administration Medical Center, Omaha, NE 68105, USA; George.Hemstreet@va.gov

**Keywords:** drug-induced nephrotoxicity, three-dimensional, organoids, chips, in vitro models, stem cells

## Abstract

The kidneys are often involved in adverse effects and toxicity caused by exposure to foreign compounds, chemicals, and drugs. Early predictions of these influences are essential to facilitate new, safe drugs to enter the market. However, in current drug treatments, drug-induced nephrotoxicity accounts for 1/4 of reported serious adverse reactions, and 1/3 of them are attributable to antibiotics. Drug-induced nephrotoxicity is driven by multiple mechanisms, including altered glomerular hemodynamics, renal tubular cytotoxicity, inflammation, crystal nephropathy, and thrombotic microangiopathy. Although the functional proteins expressed by renal tubules that mediate drug sensitivity are well known, current in vitro 2D cell models do not faithfully replicate the morphology and intact renal tubule function, and therefore, they do not replicate in vivo nephrotoxicity. The kidney is delicate and complex, consisting of a filter unit and a tubular part, which together contain more than 20 different cell types. The tubular epithelium is highly polarized, and maintaining cellular polarity is essential for the optimal function and response to environmental signals. Cell polarity depends on the communication between cells, including paracrine and autocrine signals, as well as biomechanical and chemotaxis processes. These processes affect kidney cell proliferation, migration, and differentiation. For drug disposal research, the microenvironment is essential for predicting toxic reactions. This article reviews the mechanism of drug-induced kidney injury, the types of nephrotoxicity models (in vivo and in vitro models), and the research progress related to drug-induced nephrotoxicity in three-dimensional (3D) cellular culture models.

## 1. Drug-Induced Nephrotoxicity

Chronic kidney disease affects 8–16% of the global population [1]. It is characterized by the gradual loss of key functions over time, eventually leading to kidney failure requiring dialysis or kidney transplantation to maintain life [2]. Moreover, recent studies further reveal that acute kidney injury (AKI) is positively associated with the risk of chronic kidney disease, and patients with chronic kidney disease complicated with AKI have a higher mortality rate [3]. Even with the advancement of medical technology, the incidence of AKI has gradually increased in recent years, leading to an increase in patient mortality [4]. A variety of risk factors may contribute to the occurrence of AKI, including food preparations, drugs, infection, ischemia, sepsis, and intravenous contrast agents [5,6]. In particular, drug-induced nephrotoxicity is a major contributing factor in approximately 60% of AKI cases in hospitalized patients [7].

Drug-induced nephrotoxicity causes more than 1.5 million adverse events in the United States each year, affecting approximately 20% of the adult population [8,9]. Although kidney damage is usually reversible, it is estimated that the annual management cost is approximately $3.5 billion [8]. Drug-induced nephrotoxicity is driven by multiple mechanisms, including renal tubular cytotoxicity, altered glomerular hemodynamics, inflammation, crystal nephropathy, and thrombotic microangiopathy [10,11,12].

Direct nephron-toxicant mechanisms have been most extensively studied on the renal proximal tubule epithelial cells (RPTEC) (Table 1). However, renal tubular epithelial cells express a wide range of transporters, many of which are unique to specific segments of renal tubules. Consequently, drugs with an affinity for these transporters cause cell apoptosis or death in specific nephron fractions [13,14,15]. In contrast, some drugs, such as amphotericin B, cause renal tubular toxicity by non-specifically destroying the entire tubular epithelial cell membrane. In addition, renal tubular epithelial cells may be damaged by drug penetration, resulting in drug-induced kidney stones or drug-induced ischemic events. For example, contrast agents used in radiographic procedures such as angiography cause nephropathy by inducing oxidative stress and osmosis as well as hemodynamic changes [16].

Podocytes maintain the filtration barrier in the glomerulus and are also the target of drug-induced nephrotoxicity [45]. Certain drugs have a direct toxic effect on podocytes. For example, puromycin is taken up by podocytes through the plasma membrane monoamine transporter (PMAT; also known as ENT4, encoded by SLC29A4), in contrast to bisphosphonates that cause podocyte injury by destroying the cytoskeleton [46]. Once injured, the podocytes undergo a dedifferentiation process, destroying the glomerular filtration barrier and leading to nephrotic syndrome. The resulting proteinuria can cause secondary renal tubular damage [47].

Induction of inflammatory response in the glomerulus, renal tubular epithelial cells, and surrounding stromal tissue is another drug-induced nephrotoxic mechanism, leading to renal fibrosis or scarring [48]. Glomerulonephritis is an immune-mediated inflammatory renal disorder induced by several drugs (e.g., penicillin and non-steroidal anti-inflammatory drugs) and infectious agents and is associated with proteinuria [49]. Another inflammatory disease, acute interstitial nephritis (AIN), may be an adverse reaction to several drugs such as antibiotics, phenytoin, proton pump inhibitors, allopurinol, lithium, and antiviral drugs, inducing inflammatory, non-dose-dependent responses by causing immunoglobulin deposition in tubule basement membranes [50].

Various drugs and their derivatives are insoluble in urine, which may cause crystals to precipitate in the distal renal tubule lumen, thereby restricting urine flow and triggering cellular reactions in the interstitial sites. Renal insufficiency and insufficient vascular volume increase the risk of crystal nephropathy. The urine drug concentration and the urine pH may affect the precipitation and volume “replenishment of crystals” caused by incompletely soluble drugs. Drugs associated with crystal nephropathy include antiviral drugs (i.e., indinavir and acyclovir) and anticancer agents (methotrexate) [51,52].

The kidneys also harbor a highly diverse endothelial cell population and a microvascular component. Endothelial cells in glomerular and inter-renal vessels are also sensitive to drug-induced damage. Unlike renal tubules, these endothelial cells lack regenerative capacity [53]; therefore, acute damage to the renal vascular system increases patients’ susceptibility to chronic kidney disease [54]. Nephrotoxic agents also directly affect vascular reactivity by regulating endothelial barrier function, coagulation cascade reaction, and/or inflammatory process [55]. Tubular vascular crosstalk occurs through vascular endothelial growth factor A (VEGFA) and its receptor VEGF receptor 2 (VEGFR2) expressed in renal tubular epithelial cells, which are almost completely expressed on peritubular capillary endothelial cells [56]. This interaction is essential for maintaining the peritubular microvascular system. Anticancer drugs targeting the VEGF pathway induce thrombotic microangiopathy, proteinuria, and hypertension [57]. Thrombotic microangiopathies—including thrombocytopenia, microangiopathic hemolytic anemia, and microvascular occlusion—may be immune-mediated and usually result in acute tubular necrosis. Drugs associated with thrombotic microangiopathy include quinine, cyclosporine, and tacrolimus [58].

## 2. Treatment of Drug-Induced Nephrotoxicity

The management of acute drug-induced nephrotoxicity usually involves patient hospitalization. At this stage, the overall goal is to maintain renal perfusion to ensure that the kidneys have sufficient blood/oxygen supply and restore their normal functions in time. Failure to implement appropriate measures early may ultimately lead to end-stage renal disease (ESRD) or even death.

Hypovolemia is a major risk factor for drug-induced nephrotoxicity. Therefore, blood volume and hemodynamics stabilization are critical. The choice of exchange fluid is predicated on normalizing physiological conditions. For example, in the case of insufficient vascular volume, isotonic solutions (such as saline) are recommended for fluid resuscitation, rather than hypertonic solutions (such as starch and albumin) [59] or protein. In the case of vasomotor shock, vasopressors such as norepinephrine can also be prescribed to maintain blood pressure [60].

When necessary, nutritional status and blood sugar levels should be observed and maintained. Therefore, individualized treatment is more helpful than standard procedures. Once the clinical features of nephrotoxicity are discovered, it is recommended to stop the use of nephrotoxic drugs immediately. Such methods are particularly effective for highly nephrotoxic drugs such as cisplatin, aminoglycosides, or amphotericin B [61]. According to Kidney Disease: Improving Global Outcomes (KDIGO) criteria [62], depending on the stage of kidney injury, acute nephrotoxicity can lead to the need for renal replacement therapy [63].

The steps implemented to prevent drug-induced nephrotoxicity are usually limited. Certain patient groups are more prone to drug-induced nephrotoxicity than others. Therefore, understanding the risk factors related to specific patient populations and specific drug categories in advance, combined with early diagnosis, dose-adjusted therapeutic drug monitoring, and timely prospective treatment is essential for the prevention and management of drug-induced renal damage [48].

## 3. Existing In Vitro Models for Drug-Induced Nephrotoxicity Testing

### 3.1. Two-Dimensional Culture

Traditionally, two-dimensional (2D) cell cultures are most used as in vitro cell-based studies, which represents a low-cost and robust model in achieving high-throughput screening and well-controlled experimental design. However, the cultured kidney cells on a monolayer in tissue culture plastic containers is not consistent with the in vivo microenvironment. Cells lose important interactions with extracellular matrix proteins and intercellular signals from heterogeneous cell types necessary for normal cellular responses. In addition, 2D culture represents a static model, which becomes less physiological as nutrients are consumed and metabolic waste accumulates.

Primary human renal cells are used because they more closely mimic the renal physiological state. However, these cells have limited growth capacity and tend to lose their original phenotype over time. Despite these limitations, renal cells are still a reliable choice for studying basic renal cell functions and the effects of nephrotoxic agents. To overcome the limitations of culturing primary cells, immortalized cells are frequently employed because they grow and divide indefinitely. The disadvantages of these cells include that the immortalization process itself may cause some changes over time, which can alter the cell’s functional characteristics [64].

Despite attempts to improve 2D culture conditions [65,66], the validity of 2D in vitro studies is still questionable. Except for culture conditions that are far from the highly complex in vivo conditions, the measurable toxicity endpoints in these reflect a low-complexity system, but they faithfully reflect cell viability and proliferation, have poor physiological or clinical relevance, and there is no predictable in vivo drug response [67].

### 3.2. Rodent Experiments

The use and outcome of animal models is essential to bridge the translational gap from the in vitro to the clinic. However, animal models are expensive to use, are time-consuming, require expertise, have low throughput potential, and have ethical issues, but most importantly, these models are usually less relevant to human systems [68]. This mismatch between animal and human results is mainly due to the many differences in the expression of drug transporters and metabolic enzymes between species.

Rodents and rabbits are most used as animal models to test nephrotoxicity. However, the expression levels of organic cation transporter 1 (OCT1) and OCT2 are comparable in rodent kidneys, while OCT2 is dominant in human kidneys. The advantage of rabbit is that it is a suitable animal model between rodents and larger animal models (such as primates). The rabbit size allows off-the-shelf blood sampling and makes it easier to obtain many cells and tissues from a single animal. In addition, rabbits live longer than rodents. Genetically, the rabbit immune system and human immune system are significantly more similar than rodent genomes [69]. The differences in the expression of transporters between animals and humans and between different non-human species limit the utility of animal models to study adverse drug reactions. Although these issues are improved, the development of more appropriate in vitro 3D models is needed for preclinical and early-stage clinical development [70].

## 4. Three-Dimensional Renal Culture Models for Predicting Nephrotoxicity

**Figure 1:**
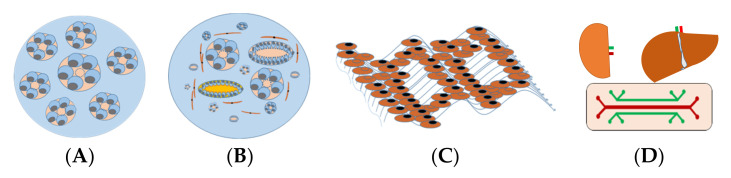
In vitro 3D kidney models for predicting nephrotoxicity. (**A**) Renal spheroids are often considered as RPTEC embedded in hydrogel to form hollow spherical cysts with an apical membrane facing the renal tubular lumen. (**B**) Organoids consist of multiple cells, different types of renal tubular, endothelial, and interstitial cells that self-organize in response to developmental cues and overcome the cellular simplicity of 2D cultures. (**C**) Three-dimensional (3D)-engineered kidney tissue consists of various renal cells with ECM as a complex and highly charged network (i.e., collagen, elastin, laminin, and glycoproteins), providing a 3D structure for the spatial organization of cells. (**D**) A kidney-liver-on-a-chip that comprises a perfusable, convoluted 3D renal tubule, and liver cells within the ECM enable fluid flow and the administration of test compounds to the apical surface of the cells.

The perception of in vitro 3D renal models is based on the creation of renal structures mimicking the physical and biochemical features of in vivo renal tissue with multiple cell types contacted to renal extracellular matrix ECM. Thus, 3D renal models are often divided into spheroids, organoids, and tissue-engineered models, and organ-on-chip models used in drug development or renal cancer modeling, are presented in Figure 1.

The average in vivo cell density is up to 7.5 × 10^7^ cells/mL, with solute concentrations of 30–80 g/L. By contrast, an in vitro 2D culture can only provide a maximum density of 10^6^ cells/mL with solute concentrations of about 1–10 g/L. Two-dimensional (2D) cell cultures lack in vivo features such as cell–cell and cell–ECM interactions, matrix chemical composition and mechanics, chemotaxis gradients of soluble cell signals, cell oxygenation, and 3D matrix structure. These limitations in 2D culture will affect cell proliferation, polarization, migration, signal transduction, and gene expression [71].

The difference in the physical and physiological properties between 2D and 3D cultures makes 2D cells more susceptible to drugs than 3D cells, because 2D cells cannot maintain their normal morphology compared to 3D cells [72]. Another reason that 2D cells respond differently to drugs than 3D cells is the cell surface receptor organization. Drugs usually target certain receptors on the cell surface. The difference in the structure and spatial arrangement of the surface receptors may affect the drug receptor binding efficiency, thereby triggering different responses [73]. Third, cells cultured in 2D are usually at the same cell proliferation stage, while 3D cells are usually at a different cell proliferation stage, similar to cells in the body [72]. In 3D cellular systems, the cell proliferation stage is limited, which is similar to the in vivo situation [74].

Microfluidics, which is a study of fluid flow in micron-size domains, proves to be an effective technology in the study both in vivo and in vitro. The capability of microfluidic devices to integrate all the necessary components in a less than a 1-inch silicon chip along with the advances in micro-electro-mechanical systems (MEMS) led to highly efficient lab-on-a-chip devices [75]. Other unique features of a microfluidic platform such as its perfect length scale fitting at cellular and tissue levels as well as a very small number of required agents make them an excellent choice for biological applications [76]. The collaboration between engineers, biologists, and medical doctors led to the advent of the organ-on-a-chip [77,78,79].

The initial design of a published kidney-on-a-chip has two compartments [80,81]. A top channel mimics the urinary lumen with fluid flow, whereas the bottom chamber mimics interstitial space and is filled with media. Kidney cells are under much lower shear stress than the endothelial cells. This device used rat distal tubular cells or Madin-Darby Canine Kidney (MDCK) cells, and its shear stress was ≈1 dyn/cm^2^ [80]. A second report [81] showed a similar design but with human proximal tubular cells attached. In a human renal cell model, the authors reproduced cisplatin nephrotoxicity in this channel system. Proximal tubular epithelial cells have much lower shear stress: ≈0.2 dyn/cm^2^ [81]. The foot processes of podocyte, a glomerular visceral epithelial cell, form a size- and charge-selective barrier to plasma protein, and derangement of the barrier causes podocyte injury and proteinuria [82]. In addition, podocyte-on-a-chip has been tried with no success yet [83]. The challenge is that podocytes are exposed under a very low shear stress in vivo and require a sophisticated culturing condition.

Although the obvious advantages of 3D culture have been demonstrated, 3D cell culture is not as widely accepted as 2D culture in the research field, which is predicated on the large structural deviation in cellular phenotypes in the 3D model. The inconsistency between the models hinders reproducible experimental data and proper system analysis. Another practical consideration hindering the widespread use of 3D models is the high price, which further limits the feasibility of large-scale experiments [72]. Table 2 summarizes the characteristics of 2D cell culture and 3D cell culture models.

## 5. In Vitro 3D Kidney Models

### 5.1. Spheroids

Renal spheroids are often considered as in vitro 3D renal tubular structures with RPTEC embedded in ECM (i.e., collagen and laminin) form structures that form hollow spherical cysts with an apical membrane facing the surrounding medium (Figure 1). Most cell types used for spheroids are human renal cell lines (Table 2). The size of a spherical cyst ranges from 150 to 350 μm with cell polarity with a cyst. In addition, the basolateral side of the cell is exposed to hypoxia, and access to this side is limited. Although protein expression can be assessed at the mRNA and protein levels, the ability to track drugs in this system can be challenging [84,85].

### 5.2. Organoids

Organoids are developed from several types of stem cells from different sources, including human embryonic stem cells (ESC), human-induced pluripotent stem cells (h-iPSC), and renal stem cells/progenitors (Table 2). Due to ethical concerns regarding the culture of embryonic stem cells, iPSC and renal stem cells are preferred. Using h-iPSC, it is possible to generate kidney organoids that contain cell types from different nephron segments. Morizane et al. established a chemically defined protocol to differentiate h-iPSC into pluripotent nephron progenitor cells (NPC), which can form organoids containing podocytes, proximal tubules, Henle rings, and distal tubules (Figure 1) based on the expressed markers. H-iPSC are sandwiched between two Matrigel layers that reduce growth factors, forming an h-iPSC sphere with a cavity [18]. Freedman et al. have shown that the inhibition of glycogen synthase kinase 3b (GSK3b) transforms h-iPSC spheres into complex tubular organoids composed of proximal tubules, endothelial cells, and podocytes [86]. Thus, renal organoids could be a suitable unlimited source of H-iPSC-derived primary proximal tubule cells. However, the characteristics of the proximal tubules of this organoid still require further study. In addition, 3D toxicity models of other key cells are lacking. Each renal cell model (organoid of podocyte, Henle, or distal cells) is desired to establish potential tools to study the role of podocytes in supporting glomerular filtration, the role of Henle and distal cells in reabsorbing water and ions, and the related drug-induced renal injuries.

Although organoids are a relatively simple method for deriving and co-cultivating various kidney cell types, the presence of different nephron segments should detect segment-specific toxicity during the screening process. The major challenge in developing renal organoids is that few biomarkers are both specific and sensitive enough for cell identification and the functional distinguishing of each renal cell type. Although there are many renal biomarkers available (Table 3), more specific renal biomarkers are needed to recognize each type of renal cells within organoids. The universal renal biomarkers for different species and humans are desirable for both experimental and clinical examples. In addition, cell type-specific damage is also important for defining the pharmacology of test compounds with 3D models, but this model has not been presented yet. Furthermore, there is a lack of organoid protocols available to produce histological and functional nephron organoids with renal corpuscle, renal tubule, and collecting ducts that merge into a single collecting system. Finally, the tubules in the organoids can hardly be perfused directionally, which limits test compound administration [70]. Thus, current organoid models can barely show how kidney cells react in an environment with fluid shear stress resembling the urinary flow in the kidney tubules [87].

### 5.3. Three-Dimensional (3D) Tissue-Engineered Kidney Model

To provide an in vitro 3D kidney-tissue model, nothing more than the kidney itself can provide a native 3D biological scaffold containing a tubular architecture and all the ECM composition, which can be achieved by decellularization of the kidney and the growing cells in it. However, the diffusion of gas and nutrition are the limiting factors for maintenance of the organ. To solve this problem, Finesilver et al. have developed a 300 μm acellular kidney fragment using a rat kidney on which HK-2 cells were grown. The kidney-derived acellular matrix consisted of different types of proteins (such as collagen types I, III, V, VI, VII, and XV), both sulfated and nonsulfated glycosaminoglycans (GAGs), glycoproteins, and polysaccharides [96]. The ECM compounds promote cell–cell and cell–ECM interactions [97] to form renal tissues. Bonandrini’s group demonstrated that rat kidneys were efficiently decellularized to produce renal ECM scaffolds and rapid recellularization of vascular structures and glomeruli using embryonic stem cells [98]. In addition, the kidney of the Rhesus monkey has been also used as a decellularized scaffold for human PSC cells to investigate the impact of the kidney scaffold on the differentiation of renal cells from PSC cells [99]. Uzarski et al. has perfused the decellularized rat kidney with human renal cortical epithelial cells to develop a kidney in a bioreactor. In these types of systems, the recellularized kidney is connected to the flow via a renal vein and ureter, and perfusion through the vein provides necessary nutrients for the cells [100]. However, the larger the kidney is in size, the more cells are required. The application of animal kidney to gain a renal scaffold and recellularization with human cells might improve a native formation of different kidney sections; however, how these sections or a whole recellularized kidney can be used for different endpoints requires further study.

Hydrogels are being investigated as scaffold biomaterials in renal tissue engineering because they resemble natural soft tissue due to their high water content contributing to biocompatible properties [101]. In addition, the high porosity of hydrogels with a polymer network is beneficial for the exchange of nutrients, gasses, waste products, and signaling molecules with the embedded cells. Many biopolymers are enzymatically degradable in vivo, with most retaining these properties even when chemically altered and crosslinked to produce a hydrogel network [102]. Synthetic hydrogels can be modified to slowly hydrolyze under physiological conditions. Thereby, cells can actively reshape their surroundings, while low molecular weight waste products are safely removed from the body. The nature of the targeted cell environment can be mimicked further through utilizing ECM-based hydrogels or by incorporating biologically relevant cues, e.g., tissue-specific growth factors [103]. A representative example is given by the Khademhosseini group, who used a commercially available steel needle embedded into a gelatin methacrylate scaffold, which was removed after gelation to form a tunnel in the hydrogel with a diameter equal to the outer diameter of the needle [104]. The surface of these tunnels was used for cell seeding via perfusion with a cell suspension. This method has recently been applied in kidney-on-chip developments [105].

### 5.4. Kidney-on-Chips

In order to further improve in vitro renal models, the implementation of fluid flow and vasculature is crucial not only to increase kidney cell maturity but also provide an environment closer to the in vivo milieu [106]. The flow rate in the lumen varies from 0.2 to 2.0 dyne/cm^2^ and triggers mechanically sensitive pathways through microvilli, primary cilia, and glycocalyx, all of which are expressed on the tubular apical membrane [107,108]. Advances in MEMS have enabled researchers to apply microfluidics to cell and vascular culture, resulting in so-called “kidneys-on-a-chip” [109]. Vriend et al. demonstrated that an immortalized proximal tubule cell line (ciPTEC) equipped with organic anion transporter 1 (OAT1) to a panel of selected drugs (ciPTEC-OAT1) can be cultivated in OrganoPlate, which is a 3D platform composed of 96 chips. A rocker plate platform provides fluid shear stress and high-throughput screening compatible with advanced imaging technology [110]. Following a similar approach, Schutgens et al. developed a new microfluidic in vitro system supporting renal tubular epithelial organoids or “tubular bodies” derived from adult stem cells. The resulting tubules show active transepithelial transport and are used to simulate a variety of diseases, including BK virus (i.e., polyomavirus), Wilms tumor, and cystic fibrosis. These results demonstrate the utility of this culture system for disease modeling or disease screening [89] and potentially for renal toxicity testing.

The liver-kidney-on-chip is another model to study drug metabolites [111]. This optimized microfluidic chip with interconnected compartments, liver-kidney-on-chip, provides the possibility of representing the exchange between liver and renal cell types and enables studying interdependent cellular responses between liver and kidneys (Figure 1). Theobald et al. demonstrated that this cycle system enhances the efficiency of toxicity analyses. In a streamlined liver-kidney-on-chip platform, hepatic cells grow in microfluidic conditions abundantly and stably expressed metabolism-related biomarkers. Toxicity and metabolic response to drugs are well evaluated in a flow-dependent manner in this system, suggesting the importance of advanced interconnected liver and kidney in microfluidic devices for application in in vitro toxicity testing and as optimized tissue culture systems for in vitro drug screening [111].

In summary, these findings indicate that the development and use of advanced in vitro 3D models of kidney, kidney–liver, or kidney with multiple organ models is an important improvement in the safety assessment of new drugs to predict nephrotoxicity [112].

## 6. Cell Types for 3D Culture Models

### 6.1. Animal Primary Renal Cells

Animal primary renal cells are an alternative cell source to develop a long-term 3D culture model of proximal renal tubules (Table 2), which can be used for in vitro nephrotoxicity studies [25]. Proximal tubules isolated from mouse kidneys were encapsulated in selected hyaluronic acid (HA)-based hydrogels, and cell survival was monitored for up to 6 weeks. The morphology, function, and physiology of epithelial cells in the HA gel culture system were evaluated. The culture response to nephrotoxin was also studied, including biomarker induction (CYP450 and KIM-1), drug metabolites, and cytokine production. In addition, the same research team also conducted a comparative study between the kidney 3D culture system and standard immortalized 2D cell lines (LLC-PK1 and HEK293) [67]. They also evaluated and compared cytokine release, renal biomarker expression, cytochrome enzyme, and cyto-kinase shedding in vivo, 3D culture, and traditional 2D culture models. The 3D model showed similar characteristics to its in vivo model, which was not observed in 2D kidney cell cultures.

### 6.2. Human-Derived Immortalized Cells Lines

Several renal cell lines are often used for 3D renal tubule spheroid models to test the drug-induced toxicity, although human and animal primary renal cells have been reported (Table 4).

HEK293 cells are a commonly used human renal cell line derived from human embryonic kidneys that shows potent differentiation plasticity. This cell line forms glomeruli, proximal convoluted tubules, loops of Henle, and distal convoluted tubule nephron markers when cultured in spheroids for 5 to 10 days [130]. Prange et al. compared a spherical cyst obtained from HK2 and cryopreserved RPTEC using hanging drop plates. These RPTEC were differentiated into spherical cysts with a luminal structure and retain AQP1, megalin, cubilin, microvilli, and tight junctions [131,132].

DesRochers et al. used hTERT immortalized human renal cortex (NKi-2) cells to design a 3D model for assaying nephrotoxicity. Compared with 2D cell culture, the 3D model that uses NKi-2 aggregation is more sensitive to the nephrotoxin and is useful for monitoring chronic injury when repeatedly dosed for up to 2 weeks, which is a limitation of traditional 2D culture methods [19]. RPTEC/TERT1 cells also self-assemble in Matrigel to form a highly differentiated and stable 3D tubular structure characterized by a branched network of monolayer cells surrounding the cell-free cavity, thereby simulating proximal tubules. This model was used to evaluate delayed cisplatin-induced nephrotoxicity and demonstrated higher sensitivity compared to 2D culture [133].

Based on these 3D culture systems, a bioengineered renal tubule was developed to simulate the physiological nephron segment geometry. In short, cipTEC-OAT1 cells were seeded on a polyethersulfone (PES) hollow fiber membrane pretreated with 3,4-dihydroxy-1-phenylalanine (l-DOPA) and collagen IV A double coating. Using this system, the excretion of protein-bound uremic toxins and the reabsorption of albumin under perfusion conditions simulate the renal native environment and portray remote sensing and signaling pathways that balance the level of microbial metabolites in the human body [134]. In addition, vitamin D-activated bioengineered renal tubules sense the damage induced by uremic toxins. The use of decellularized kidneys is also a suitable platform for investigating drug-induced nephrotoxicity. Fedecostante et al. used sodium dodecyl sulfate (SDS) to decellularize excess rat kidneys and recellularized these kidneys with ciPTEC-OAT1. Compared with the 2D system, the model had increased sensitivity to cisplatin, tenofovir, and cyclosporin A [135].

### 6.3. Animal Immortalized Cells Lines

MDCK cells are a model canine cell line used in drug screening and biomedical research [136]. In the presence of hepatocyte growth factor, spherical cysts that MDCK cells produced are stimulated to form long tubules [137]. Spherical encapsulations can also be obtained by culturing cells in hanging drop plates and rotating wall containers on low-attachment surfaces coated with 0.5% agarose medium and covered with phosphate-buffered saline (PBS) on top [138]. Pig Kidney Epithelial cells (LLC-PK1) is a cell line derived from the kidney of a normal, healthy male pig (Sus scrofa) [139]. Terashima et al. used LLC-PK1 cells on the synthetic membrane device, but the exposure to blood urea nitrogen (BUN) and creatinine and their multi-layer growth on the device limits clinical utility [140]. In addition to the symmetric hollow fiber membrane, Ueda et al. introduced an asymmetric membrane with hemo-compatible and cyto-compatible surfaces. In this device, MDCK and LLC-PK1 cells formed confluent monolayers on the more adhesive cyto-compatible surface [141,142].

### 6.4. Human ESC/Human iPSC

To date, the kidney organoids produced represent early developing nephrons but lack the functionality of mature epithelia [22]. However, the proximal tubules in kidney organs developed by other groups maintained characteristic functions of mature tubules. Organoids produced by Takasato regenerate proximal tubules that take up dextran. Compared with other cell types in organoids, organoids are more sensitive to cisplatin-induced apoptosis, although expression to other injury markers has not been determined. Morizane et al. developed organoids using different protocols in which the proximal tubules express KIM1 in response to cisplatin and gentamicin and demonstrated that cisplatin induces specific DNA damage in the proximal tubules, similar to phosphorylated histone H2AX (γH2AX) assayed by [18]. In addition, Chuva et al. developed a two-step model to transplant kidney organoids into the chorioallantoic membrane of chickens to provide a vascularized environment to promote organoid maturation [143]. These findings indicate that organics may provide a nephrotoxicity screening platform, but it remains necessary to further describe the functional maturity and utility for monitoring known nephrotoxic drugs.

### 6.5. Human Urine-Derived Stem Cells

Urine-derived stem cells (USC) have the required regenerative properties, including strong proliferation potential, pluripotent differentiation, and paracrine effects [122,123,124]. USC have been successfully induced to differentiate into urothelium [144]. In addition, USC have been applied to the personalized modeling of inherited kidney disease and motor neuron disease. Compared with adult stem cells and h-iPSC, USC can be obtained noninvasively at a lower cost and with a simpler method. These encouraging findings support the possibility of generating organoids from USC and their subsequent application for drug screening.

**Figure 2:**
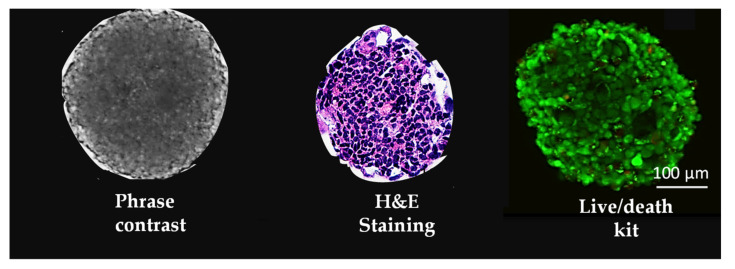
Three-dimensional (3D) renal organoids of human urine-derived stem cells (USC). Renal tubular organoids (4 × 10^3^ cells, 338 ± 10 μm at diameter) have been developed with human USC combined with the pig-derived kECM system to verify renal tubular-like structures located along with the outer surface of 3D spheroids one week after culture.

USC organoids (Figure 2) have been developed using the pig-derived kECM system to verify kidney function [28]. Human USC differentiates into podocyte-like cells in 3D organoids and forms renal tubular-like structures. In addition, using differentiated USC organoids treated with cisplatin (0.2 mM) or acetone (1%) for 72 h, as assessed by organoid morphology and the expression of KIM-1 and CYP450, a loss of semi-transparency at the edge and cloudy organoid centers are indicative of dead cells. H&E-stained sections confirmed this, showing that the volume of apoptotic cells decreased, leading to an increase in the nuclear/cytoplasmic ratio. Moreover, the expression of CYP450 and KIM-1 increased, indicating that organoids are sensitive to nephrotoxic drugs cisplatin and acetone, proving that USC organoids are a promising screening tool for drug nephrotoxicity [28].

## 7. Methods to Induce Stem Cells to Give Rise to Renal Cells

In 2014, Taguchi et al. used mouse cells to construct an in vitro differentiation protocol for kidney organoids for the first time. This program successfully differentiated iPSC into metanephric mesenchyme cells and redefines the kidney differentiation process. Metanephric mesenchyme cells finally differentiate into kidney organoids with renal tubules and glomeruli through the regulation of the classic Wnt pathway [88,145]. This protocol is a milestone in the study of kidney organoid differentiation. Takasato et al. constructed a differentiation protocol that induces the differentiation of ureteric bud and metanephric mesenchyme simultaneously in vitro [22,120]. Compared with Taguchi’s protocol, the new plan was finally successfully differentiated into the renal unit connected to the collecting duct, which contains proximal tubular epithelial cells, distal tubular epithelial cells, and glomerular structures. Although this protocol can differentiate metanephric mesenchyme and ureteric bud together, it does not form a mature kidney unit.

In 2015, Freedman et al. established the “sandwich” culture method, which refers to, in addition to the underlying Matrigel, a higher concentration of Matrigel covered on the stem cells to form a special “sandwich” structure of “Matrigel–h-iPSC–Matrigel” [86]. Compared with other schemes, this scheme has simple steps and low cost, but because only one induction reagent is used, there are more off-target differentiated cells [146]. Morizane et al. established a scheme that can construct kidney organoids in 2D and 3D culture environments and can differentiate NPCs with a high efficiency of 75% to 92% [18]. Compared with Takasato’s protocol, this protocol differentiated cells with higher maturity.

Although the kidney organoids obtained in the above protocol contain the main kidney cell types, the structure is very different from that of a normal kidney. To solve this problem, Taguchi and Nishinakamura constructed a plan to build a kidney organ similar to a normal kidney structure. This kidney organ is called a Higher-Order Kidney organ. Taguchi believes that after induced ureteric bud and induced nephron progenitor are co-cultured, the two will produce some shared transcription factors during their development, which influence each other to promote cell differentiation and maturation. Taguchi’s co-culture method forms a kidney unit with a ureter, opening up a new kidney organoid culture [147]. In 2019, Low et al. discovered a protocol to differentiate vascularized kidney organoids. This method can effectively vascularize kidney organs and also provides a new idea for the source of blood vessels in the kidneys [94,148].

Recently, Guo et al. used USC to generate 3D human renal tubular organoids for nephrotoxicity screening [26]. They centrifuged fresh urine samples from healthy male individuals at 500 g for 5 min. After discarding the urine supernatant, cell pellets were gently suspended in the USC culture medium consisting of an embryo fibroblast medium (EFM) and keratinocyte serum-free medium (KSFM) mixed at a ratio of 1:1 with 10% fetal bovine serum (FBS). Then, the cells were cultured in 24-well plates at 37 °C in a 20% O_2_/5% CO_2_ incubator. After 3–5 days, USC individual clones appeared, which was considered as passage 0 (*p*0). Each clone was trypsinized, and the culture medium was changed. When reaching a confluence of 60–70%, the cells were sub-cultured and re-plated into 6-well plates (*p*1). USC at p3 were resuspended in medium and seeded into a 96-well Clear Round Bottom Ultra-Low Attachment Microplate at 37 °C in an atmosphere of 5% CO_2_. The organoids were maintained in culture for 7 days. Half of the culture medium was removed and replaced with fresh medium every day. To induce USC differentiation into renal cells, 1 μg/mL solubilized porcine k-ECM was added into the culture medium at a ratio of 9:1 (culture medium: k-ECM gel) for 14 days. Table 5 shows various methods used to induce the reno-differentiation of stem cells.

## 8. Procedures to Set Up 3D Models for Drug-Induced Nephrotoxicity Testing

In 2012, Astashkina et al. assessed the ability of kidney proximal tubules 3D organoids to up-regulate CYP2E1 enzymes and Kim-1 protein response to well-known nephrotoxins (acetone, cisplatin). Three-dimensional (3D) organoid cultures were assessed for their capacity to induce pro-inflammatory events associated with toxicity, leading to acute kidney injury (AKI) [25]. DesRochers et al. used immortalized human renal cortex epithelial cells to bioengineer a 3D kidney tissue model and validated the model using biomarkers including LDH Kim-1 and NGA. They compared acute (3 days) and chronic (2 weeks) toxicity induced by cisplatin, gentamicin, and doxorubicin. The comparison confirmed that 3D tissues were more sensitive to drug-induced toxicity and, unlike 2D cells, were more predictive for monitoring chronic toxicity [19]. An in vitro 3D micro-physiological system developed by Adler was employed to evaluate cadmium chloride toxicity. After challenging the kidney-on-a-chip with cadmium chloride for 48 h, both Kim-1 and HO-1 increased to varying degrees [40]. Fedecostante et al. developed a new three-dimensional (3D) nephrotoxicity platform based on a decellularized rat kidney scaffold, which was recellularized with conditionally immortalized human RPTEC overexpressing the organic anion transporter 1 (ciPTEC–OAT1). Compared with 2D culture, recellularized scaffolds were more sensitive to cisplatin, tenofovir, and cyclosporine toxicity after 24 h of exposure [26].

Takasato et al. treated kidney organoids with cisplatin (0.5–20 mM) for 24 h before quantifying cleaved caspase 3 antibody-staining. While control organoids showed occasional apoptotic interstitial cells, both 5 mM and 20 mM cisplatin induced specific acute apoptosis in mature proximal tubular cells, whereas immature cells did not undergo apoptosis [22]. Morizane et al. used gentamicin (5 mg/mL) and cisplatin (5 μM) to treat kidney organoids derived from 3D h-iPSC for 48 h and 24 h, respectively. Staining of whole and frozen sections of organoids treated with gentamicin showed that there was obvious KIM-1 expression on the lumen surface of LTL^+^ tubules but not in Cadherin 1^+^ tubules. Real-time PCR confirmed gentamicin dose-dependent up-regulation of KIM-1 by the hormone, indicating that gentamicin damaged the proximal tubules in the organoids. In addition, cisplatin significantly up-regulates KIM-1 in LTL^+^ tubules and inhibits Cadherin 1^+^ expression, indicating proximal and distal renal tubular toxicity [26].

Musah et al. used microfluidic glomerular chips arranged by h-iPSC-derived podocytes and glomerular endothelial cells to simulate kidney injury. To test this possibility, they exposed the glomerular chip to the anticancer drug doxorubicin for 5 days, which was continuously infused through a blood vessel lining the endothelial channel. Microscopic imaging showed a dose-dependent destruction of the podocyte layer and cell detachment in the urinary channel. The quantification of phalloidin-stained cells confirmed the obvious dose-dependent stratification of doxorubicin-treated podocytes from the flexible ECM coating that separates the urinary and vascular channels [43].

Bajaj et al. differentiated h-iPSC into 3D multicellular structures containing proximal tubule cells and podocytes and evaluated them as a platform for predicting nephrotoxicity. The model can correctly identify four renal tubular toxins (gentamicin, citrinin, cisplatin, and rifampicin), as evidenced by the increase in the renal tubular biomarkers KIM-1 and HO-1. When differentiated cells were treated with doxorubicin and puromycin, mainly glomerular toxins, increased levels of NPHS1/WT1 were observed [17]. Kumar et al. described a modified suspension culture method for the generation of kidney micro-organoids from h-iPSC. To evaluate the utility of micro-organoids for assessing drug toxicity, kidney micro-organoids were treated with different doses of Adriamycin for 24 h in a 24-well plate format. Podocytes within the kidney micro-organoids exhibited TUNEL positivity within the podocyte compartment after Adriamycin treatment. qPCR analysis showing dose-dependent toxicity induced by Adriamycin on kidney organoids by the reduced expression for kidney marker genes [42]. Petrosyan et al. described a glomerulus-on-a-chip (referred to as GOAC) constituted by human podocytes and human glomerular endothelial cells (hGEC) seeded on Organoplates TM (MIME-TAS). They exposed the GOAC to puromycin aminonucleoside (PAN). PAN induced podocyte injury assayed by cytoskeleton rearrangement and loss of permselectivity for albumin at 60 min after stimuli [44]. Chips generated with diseased podocyte cell lineages will help us understand the cellular and molecular mechanisms responsible for glomerular injury and podocyte loss.

A sentinel step forward would be to induce a true stromal cell lineage from h-iPSC to produce a higher-order structure such as human kidney reconstructive models. This review reflects the evidence confirming that stromal cells have an important role in kidney organogenesis [150,151,152]. Most stromal cells in the kidney are derived from Forkhead box D1 (FOXD1)-positive stromal progenitors histologically located at the periphery of the developing kidney [153,154]. Takasato’s protocol induces the development of stromal cells, some of which express FOXD1, at the same time as the development of NPCs [22]. The stromal cells in organoids generated using the Morizane’s protocol proliferate in response to IL-1β, possibly mimicking kidney fibrosis [155]. However, there are very few studies testing the nephrotoxicity of these drugs in 3D organoids.

## 9. Conclusions

Existing 2D cultures and preclinical rodent models cannot accurately predict nephrotoxicity, which highlights the need for more accurate models. In vitro 3D renal culture systems provide an alternative tool for nephrotoxicity screening. There are still challenges to the development of 3D renal organoids for nephrotoxicity, such as human primary cell sources instead of renal cell lines, the ratio of each type of renal cells within organoids, chronic renal toxicity models, coordination between in vitro models and in vivo renal tissues in responding to nephrotoxic agents, and general lack of podocyte toxicity platforms. Recently, one study reported a 3D human iPSC-derived organoid glomeruli model [95], which provides an opportunity to test drug-induced podocyte toxicity. Advances in our understanding of kidney development, the regeneration of human kidney cell types from pluripotent stem cells, and increasingly complex tissue culture 3D platforms have stimulated the development of new methods to address these challenges. Human USC offer an optimal cell source that can be obtained via noninvasive, simple, safe, and low-cost apaches. Newer models are expected to include the features mandatory for successful candidate toxicological drug testing, including 3D structural features, a combination of multiple renal cell types with k-ECM composition, and fluid flow system. The main goal will be to generate 3D renal cell models to demonstrate that functional maturity is improved, thereby refining the response and prediction of renal injury to known and new nephrotoxic substances. Further technological advances will lead to the development of models containing multiple kidney cell types and nephron segments and to be able to access the basolateral and apical parts of the model nephrons, thereby improving the model’s physiological relevance. There is also a need to improve compound throughput testing and data collection. Ultimately, the integration of the information obtained from these in vitro models into calculation algorithms containing patient-specific physiological parameters can not only reduce the late loss of drugs in the development pipeline but also promote the development of safer drugs preventing nephrotoxic side effects and improving the effectiveness of clinically important compounds.

## Figures and Tables

**Table 1:** Methods used for in vitro nephrotoxicity assessment in 3D models and in vivo renal tissues.

Methods	Renal Tubule Epithelia Cells		Podocytes Stromal Cells	
	Drugs	Targeted Cells	Drugs	Drugs
Drugs, chemicals, or toxic agents with different doses	Gentamicin [17,18,19,20] Citrinin [17,21] Cisplatin [17,18,19,22,23,24,25,26,27,28,29] Rifampicin [17,30] Acetone [25,28,31] Aspirin [24,27,32] Penicillin G [24,27,33] Tenofovir [26,34] Cyclosporin A [26,35,36] Adriamycin [19,25,37] 4-aminophenol (PAP) [25,38] Colchicine [25,39] Cadmium chloride [40,41]	Brush border membrane of the proximal tubules S2 proximal tubular segment Basolateral membrane of proximal tubules Apical membrane of renal proximal tubules S1 and S2 proximal tubular segment Loop of Henle Brush border membrane of the proximal tubules Basolateral mem-brane of proximal tubules Brush border membrane of the proximal tubules/Thick ascending limb of the loop of Henle Brush border membrane of the proximal tubules Loop of Henle S3 proximal tubular segment S1 proximal tubular segment	Doxorubicin [17,42,43] Aspirin [27] Penicillin G [27] Puromycin-aminonucleoside [17,44] Adriamycin [42,43]	Doxorubicin [17,43] Puromycin-aminonucleoside [17]
Time-frames	24 h [17,18,22,26] 48 h [18,24,27,40] 72 h [19,25,28] 2 wks [19]		60 min [44] 24 h [17,22,42] 48 h [27] 5 days [43]	24 h [17] 5 days [43]
Biomarkers				
Gene markers	Kim-1 [17,18,23,24] HO-1 [17]		NPHS1 [17,42] WT1 [17] SYNPO [42]	
Protein markers	Kim-1 [18,19,25,28,40] CYP2E1 [25,28] HO-1 [40] NGAL [19] AQP1 IL-6 [25] TNF [25] MCP-1 [25] IL-1b [25] MIP-1a [25] Rantes [25] Cleaved-caspase 3 [22]			

Abbreviations: Kim-1, kidney injury molecule-1; HO-1, heme oxygenase 1; CYP2E1, cytochrome P450 family 2 subfamily E member 1, NGAL, neutrophil gelatinase-associated lipocalin; IL, interleukin; TNF, tumor necrosis factor; MCP-1, monocyte chemotactic protein 1; MIP-1a, macrophage inflammatory protein-1a. h, Hours; wks, weeks.

**Table 2:** In vitro 2D vs. 3D renal models of drug-induced nephrotoxicity.

Models	Advantages	Disadvantages
2D culture	-Robust model -Easy to assess, manipulate -Cost- and time-efficient -Large scale -Retention of key metabolic	-Static model -Dedifferentiation -Lack of in vivo-like morphologic and phenotypic characteristics -Low complexity -Little predictive -Poor physiological or clinical relevance
3D culture	-In vivo-like cell shape -More physiologic characteristics -Response to toxic insults with biomarkers found in vivo -3D paracrine and autocrine signaling; -Potential penetration gradients toward center -Cells of different stages (proliferating, hypoxic, quiescent, and necrotic) possible -More similar to in vivo expression profiles -Better predictive values to in vivo compound responses	-Cost-intensive -Simplified architecture -Can be variable -Less amenable to HTS/HCS -Hard to reach in vivo maturity -Complication in assay -Lack vasculature -May lack key cell types
Animal models	-Physiological resemblance -Well established -Physiological relevance -Complete organism -Test drug metabolism	-Species differences -Low throughput -Poor prediction -Ethical concerns -High costs

Abbreviations: 2/3D, 2/3-dimensional; HTS/HCS, high-throughput screening/high content screening.

**Table 3:** Biomarkers of renal cells.

Renal Cell Types	Biomarkers		References
	Protein markers	m-RNA markers	
Podocytes	Wilms tumor-1 Nephrin Podocin Podocalyxin Synaptopodin	NPHS1 NPHS2 Synaptopodin Wilms tumor-1 Podocalyxin	[18] [88] [89] [23] [90]
Proximal tubules	Lotus tetragonolobus lectin Aquaporin-1 (AQP1) Cadherin 6 Jagged 1 Megalin Kidney injury molecule 1	ABCC1 ABCC3 ABCC4 SLC22A3 SLC40A1	[18] [88] [89]
Loop of Henle	Cadherin 1 Uromodulin	Claudin 10 Claudin 14 SLC12A1 Uromodulin	[18] [91] [92] [89]
Distal tubules	Pterin-4 alpha-carbinolamine dehydratase 1 Solute carrier family 41 member 3; Cadherin 1 Brn1 Na^+^/Cl^–^ cotransporter GATA Binding Protein 3	Pterin-4 alpha-carbinolamine dehydratase 1 Solute carrier family 41 member 3; Cadherin 1 SLC12A3 Calbindin 1	[18] [88] [89]
Collecting ducts	Dolichus biflorisagglutinin Aquaporin-2 (AQP2) Aquaporin-3 (AQP3)	Cadherin 1 GATA Binding Protein 3 Aquaporin-3	[23] [89]
Endothelial cell	Platelet and endothelial cell adhesion molecule 1 Cadherin 5 Fms related receptor tyrosine kinase 1 Cluster of differentiation 34 Cluster of differentiation 31	Platelet and endothelial cell adhesion molecule 1 Cadherin 5 Fms related receptor tyrosine kinase 1 CD34	[23] [93] [89] [94]
Mesangial cells	Platelet-derived growth factor receptor beta Insulin like growth factor binding protein 5 Transgelin Matrix metallopeptidase 2	Actin alpha 2, smooth muscle Collagen type I alpha 1 chain Transgelin	[23] [95]

Abbreviations: ABCC1, ATP-binding cassette sub-family C member 1; SLC22A3, Solute carrier family 22 member 3.

**Table 4:** Different cell types used in 3D culture models.

Cell Types	Advantages	Disadvantages	References
Cell lines: -HK2 -NKi-2 -ciPTEC -RPTEC/TERT1	-A proximal tubular cell (PTC) line derived from normal kidney, immortalized by transduction with human papilloma virus 16 (HPV-16) E6/E7 genes -Act as a positive control -Stable cell line -Potentially valuable in toxicity and drug transporter assays -Stable cell line-Broad transporter and metabolic enzyme expression -Polarized tight monolayer formation-Potentially valuable in toxicity and drug transporter assays	-Limited transporter or proximal tubule characteristics -Low prediction -Limited data available for in vitro to in vivo extrapolation -Limited data available for in vitro to in vivo extrapolation	[113,114] [19] [115,116,117]
Human primary renal cells: -fetal renal cells -biopsy-derived renal cells	-Complete transporter and metabolic enzyme expression -Polarized tight monolayer formation. -High predictivity -Transepithelial transport -Broad range of biomarker assays available	-Expression of relevant proteins rapidly decreased -Limiting long-term exposure -Batch-to-batch variation -Limited availability	[81,118,119]
Human stem cells: -ESC -iPSC -USC	-In vivo-like complexity -In vivo-like architecture -Contains a variety of kidney cells, including proximal and distal renal tubular cells, endothelial cells, podocytes, and kidney-derived cells for high-throughput screening -Patient specific -Robust proliferative potential -Multipotential differentiation -Paracrine effects -Renal progenitor/stem cells -Obtained noninvasively at a less cost and simpler method	-Not free from ethical and legal issues -Immaturity -Mal-differentiation to non-renal cells Cultures are sometime contaminated when urine samples are obtained from female donors	[115,120] [115,121] [122,123,124]
Animal primary renal cells -Mouse -Rat -Rabbit Animal cell lines -Dog (MDCK) -Pig (LLC-PK1) -Monkey (VERO)	-Complete transporter and metabolic enzyme expression -Polarized tight monolayer formation -Transepithelial transport -Broad range of biomarker assays available -Stable cell line -Well established -Formation of polarized tight monolayer	-Species differences -Relatively low predictivity -Animal experiments -Species differences -Low predictivity	[125,126] [127,128,129]

Abbreviations: HK2, human kidney 2; LLC-PK, proximal-like porcine kidney cells; MDCK, Madin–Darby canine kidney; Nki-2, human telomerase reverse transcriptase immortalized human renal cortical cells; RPTEC, renal proximal tubule epithelial cell; ESC, embryonic stem cells; iPSC, induced pluripotent stem cells; USC, urine-derived stem cells.

**Table 5:** Methods used to induce reno-differentiation of stem cells.

Methods	Fabrication	Mechanism and Benefits	Limitations
Conditioned medium from renal cell culture	Matrigel–Stem Cells–Matrigel [23,86]	-Form a tubular structure, which contain proximal tubules, distal tubules, and podocytes -Simple steps and low cost	-More off-target differentiated cells
Co-culture with renal cells	Induce differentiation into iUB and iNP, and then co-culture the two kinds of cells with stromal cells in the same low-adhesion 96-well plate, and induce with RA, CHIR99021, and FGF9 [147]	-Similar to normal kidney -Mutual promotion of cell differentiation and maturation	-Inefficient
Renal ECM	One gram of ECM was mixed with 100 mg of pepsin from porcine gastric mucosa and sterilized by gamma irradiation (1 Mrad). The supernatant solution was neutralized with 0.1 N NaOH and stored at −80 °C [27,28]	-Its compositional, structural, and molecular similarity to human k-ECM -Available in large amounts	-Potential loss of soluble growth factors and cytokines during the decellularization process -Heterogeneous composition of the ECM from batch to batch
Growth factors: HGF FGF9	Company name: PeproTech [18,149] R&D Systems [88] PeproTech [94]	-Precisely regulated to the post-intermediate mesoderm stage -Express Hoxd11 -Ensure differentiation into metanephric mesenchyme	-Immature renal unit -With no specific renal cell types

Abbreviations: ECM, extracellular matrix; HGF, rh-hepatocyte growth factor; FGF9, fibroblast growth factor 9; iUB, induced Ureteric Bud; iNP, induced Nephron Progenitor.

## References

[1] Jha V., Garcia-Garcia G., Iseki K., Li Z., Naicker S., Plattner B., Saran R., Wang A.Y., Yang C.W. (2013). Chronic kidney disease: Global dimension and perspectives. Lancet.

[2] Collins A.J., Foley R.N., Chavers B., Gilbertson D., Herzog C., Johansen K., Kasiske B., Kutner N., Liu J., St Peter W. (2012). United States Renal Data System 2011 Annual Data Report: Atlas of chronic kidney disease & end-stage renal disease in the United States. Am. J. Kidney Dis..

[3] Chen N., Chen X., Ding X., Teng J. (2018). Analysis of the high incidence of acute kidney injury associated with acute-on-chronic liver failure. Hepatol. Int..

[4] Singbartl K., Kellum J.A. (2012). AKI in the ICU: Definition, epidemiology, risk stratification, and outcomes. Kidney Int..

[5] Perazella M.A. (2012). Drug use and nephrotoxicity in the intensive care unit. Kidney Int..

[6] Mukherjee K., Chio T.I., Gu H., Sackett D.L., Bane S.L., Sever S. (2021). A Novel Fluorogenic Assay for the Detection of Nephrotoxin-Induced Oxidative Stress in Live Cells and Renal Tissue. ACS Sens..

[7] Khajavi Rad A., Mohebbati R., Hosseinian S. (2017). Drug-induced Nephrotoxicity and Medicinal Plants. Iran J. Kidney Dis..

[8] Davis-Ajami M.L., Fink J.C., Wu J. (2016). Nephrotoxic Medication Exposure in U.S. Adults with Predialysis Chronic Kidney Disease: Health Services Utilization and Cost Outcomes. J. Manag. Care Spec. Pharm..

[9] Faria J., Ahmed S., Gerritsen K.G.F., Mihaila S.M., Masereeuw R. (2019). Kidney-based in vitro models for drug-induced toxicity testing. Arch. Toxicol..

[10] Schetz M., Dasta J., Goldstein S., Golper T. (2005). Drug-induced acute kidney injury. Curr. Opin. Crit. Care.

[11] Pisoni R., Ruggenenti P., Remuzzi G. (2001). Drug-induced thrombotic microangiopathy: Incidence, prevention and management. Drug Saf..

[12] Medina P.J., Sipols J.M., George J.N. (2001). Drug-associated thrombotic thrombocytopenic purpura-hemolytic uremic syndrome. Curr. Opin. Hematol..

[13] Momper J.D., Nigam S.K. (2018). Developmental regulation of kidney and liver solute carrier and ATP-binding cassette drug transporters and drug metabolizing enzymes: The role of remote organ communication. Expert Opin. Drug Metab. Toxicol..

[14] Kim J.Y., Bai Y., Jayne L.A., Hector R.D., Persaud A.K., Ong S.S., Rojesh S., Raj R., Feng M., Chung S. (2020). A kinome-wide screen identifies a CDKL5-SOX9 regulatory axis in epithelial cell death and kidney injury. Nat. Commun..

[15] Sancho-Martinez S.M., Lopez-Novoa J.M., Lopez-Hernandez F.J. (2015). Pathophysiological role of different tubular epithelial cell death modes in acute kidney injury. Clin. Kidney J..

[16] Mamoulakis C., Tsarouhas K., Fragkiadoulaki I., Heretis I., Wilks M.F., Spandidos D.A., Tsitsimpikou C., Tsatsakis A. (2017). Contrast-induced nephropathy: Basic concepts, pathophysiological implications and prevention strategies. Pharmacol. Ther..

[17] Bajaj P., Rodrigues A.D., Steppan C.M., Engle S.J., Mathialagan S., Schroeter T. (2018). Human Pluripotent Stem Cell-Derived Kidney Model for Nephrotoxicity Studies. Drug Metab. Dispos..

[18] Morizane R., Lam A.Q., Freedman B.S., Kishi S., Valerius M.T., Bonventre J.V. (2015). Nephron organoids derived from human pluripotent stem cells model kidney development and injury. Nat. Biotechnol..

[19] DesRochers T.M., Suter L., Roth A., Kaplan D.L. (2013). Bioengineered 3D human kidney tissue, a platform for the determination of nephrotoxicity. PLoS ONE.

[20] Balakumar P., Rohilla A., Thangathirupathi A. (2010). Gentamicin-induced nephrotoxicity: Do we have a promising therapeutic approach to blunt it?. Pharmacol. Res..

[21] Jagdale P.R., Dev I., Ayanur A., Singh D., Arshad M., Ansari K.M. (2020). Safety evaluation of Ochratoxin A and Citrinin after 28 days repeated dose oral exposure to Wistar rats. Regul. Toxicol. Pharmacol..

[22] Takasato M., Er P.X., Chiu H.S., Maier B., Baillie G.J., Ferguson C., Parton R.G., Wolvetang E.J., Roost M.S., Chuva de Sousa Lopes S.M. (2015). Kidney organoids from human iPS cells contain multiple lineages and model human nephrogenesis. Nature.

[23] Czerniecki S.M., Cruz N.M., Harder J.L., Menon R., Annis J., Otto E.A., Gulieva R.E., Islas L.V., Kim Y.K., Tran L.M. (2018). High-Throughput Screening Enhances Kidney Organoid Differentiation from Human Pluripotent Stem Cells and Enables Automated Multidimensional Phenotyping. Cell Stem Cell.

[24] Ding B., Sun G., Liu S., Peng E., Wan M., Chen L., Jackson J., Atala A. (2020). Three-Dimensional Renal Organoids from Whole Kidney Cells: Generation, Optimization, and Potential Application in Nephrotoxicology In Vitro. Cell Transplant..

[25] Astashkina A.I., Mann B.K., Prestwich G.D., Grainger D.W. (2012). A 3-D organoid kidney culture model engineered for high-throughput nephrotoxicity assays. Biomaterials.

[26] Fedecostante M., Westphal K.G.C., Buono M.F., Sanchez Romero N., Wilmer M.J., Kerkering J., Baptista P.M., Hoenderop J.G., Masereeuw R. (2018). Recellularized Native Kidney Scaffolds as a Novel Tool in Nephrotoxicity Screening. Drug Metab. Dispos..

[27] Sun G., Ding B., Wan M., Chen L., Jackson J., Atala A. (2020). Formation and optimization of three-dimensional organoids generated from urine-derived stem cells for renal function in vitro. Stem Cell Res. Ther..

[28] Guo H., Deng N., Dou L., Ding H., Criswell T., Atala A., Furdui C.M., Zhang Y. (2020). 3-D Human Renal Tubular Organoids Generated from Urine-Derived Stem Cells for Nephrotoxicity Screening. ACS Biomater. Sci. Eng..

[29] Pabla N., Dong Z. (2008). Cisplatin nephrotoxicity: Mechanisms and renoprotective strategies. Kidney Int..

[30] Elmeliegy M., Vourvahis M., Guo C., Wang D.D. (2020). Effect of P-glycoprotein (P-gp) Inducers on Exposure of P-gp Substrates: Review of Clinical Drug-Drug Interaction Studies. Clin. Pharmacokinet..

[31] Brown E.M., Hewitt W.R. (1984). Dose-response relationships in ketone-induced potentiation of chloroform hepato- and nephrotoxicity. Toxicol. Appl. Pharmacol..

[32] Asif S., Mudassir S., Toor R.S. (2018). Histological Effects of Nigella Sativa on Aspirin-Induced Nephrotoxicity in Albino Rats. J. Coll. Physicians Surg. Pak..

[33] Imaoka T., Kusuhara H., Adachi-Akahane S., Hasegawa M., Morita N., Endou H., Sugiyama Y. (2004). The renal-specific transporter mediates facilitative transport of organic anions at the brush border membrane of mouse renal tubules. J. Am. Soc. Nephrol..

[34] Perazella M.A. (2019). Drug-induced acute kidney injury: Diverse mechanisms of tubular injury. Curr. Opin. Crit. Care.

[35] Bakker R.C., van Kooten C., van de Lagemaat-Paape M.E., Daha M.R., Paul L.C. (2002). Renal tubular epithelial cell death and cyclosporin A. Nephrol. Dial. Transplant..

[36] Betton G.R., Kenne K., Somers R., Marr A. (2005). Protein biomarkers of nephrotoxicity: A review and findings with cyclosporin A, a signal transduction kinase inhibitor and N-phenylanthranilic acid. Cancer Biomark..

[37] Li W., He W., Xia P., Sun W., Shi M., Zhou Y., Zhu W., Zhang L., Liu B., Zhu J. (2019). Total Extracts of Abelmoschus manihot L. Attenuates Adriamycin-Induced Renal Tubule Injury via Suppression of ROS-ERK1/2-Mediated NLRP3 Inflammasome Activation. Front. Pharmacol..

[38] Klos C., Koob M., Kramer C., Dekant W. (1992). p-aminophenol nephrotoxicity: Biosynthesis of toxic glutathione conjugates. Toxicol. Appl. Pharmacol..

[39] Romano G., Favret G., Catone B., Bartoli E. (2000). The effect of colchicine on proximal tubular reabsorption. Pharmacol. Res..

[40] Adler M., Ramm S., Hafner M., Muhlich J.L., Gottwald E.M., Weber E., Jaklic A., Ajay A.K., Svoboda D., Auerbach S. (2016). A Quantitative Approach to Screen for Nephrotoxic Compounds In Vitro. J. Am. Soc. Nephrol..

[41] Faiz H., Boghossian M., Martin G., Baverel G., Ferrier B., Conjard-Duplany A. (2015). Cadmium chloride inhibits lactate gluconeogenesis in mouse renal proximal tubules: An in vitro metabolomic approach with ^13^C NMR. Toxicol. Lett..

[42] Kumar S.V., Er P.X., Lawlor K.T., Motazedian A., Scurr M., Ghobrial I., Combes A.N., Zappia L., Oshlack A., Stanley E.G. (2019). Kidney micro-organoids in suspension culture as a scalable source of human pluripotent stem cell-derived kidney cells. Development.

[43] Musah S., Mammoto A., Ferrante T.C., Jeanty S.S.F., Hirano-Kobayashi M., Mammoto T., Roberts K., Chung S., Novak R., Ingram M. (2017). Mature induced-pluripotent-stem-cell-derived human podocytes reconstitute kidney glomerular-capillary-wall function on a chip. Nat. Biomed. Eng..

[44] Petrosyan A., Cravedi P., Villani V., Angeletti A., Manrique J., Renieri A., De Filippo R.E., Perin L., Da Sacco S. (2019). A glomerulus-on-a-chip to recapitulate the human glomerular filtration barrier. Nat. Commun..

[45] Paueksakon P., Fogo A.B. (2017). Drug-induced nephropathies. Histopathology.

[46] Perazella M.A., Markowitz G.S. (2008). Bisphosphonate nephrotoxicity. Kidney Int..

[47] Srivastava T., Heruth D.P., Duncan R.S., Rezaiekhaligh M.H., Garola R.E., Priya L., Zhou J., Boinpelly V.C., Novak J., Ali M.F. (2021). Transcription Factor beta-Catenin Plays a Key Role in Fluid Flow Shear Stress-Mediated Glomerular Injury in Solitary Kidney. Cells.

[48] Naughton C.A. (2008). Drug-induced nephrotoxicity. Am. Fam. Physician.

[49] Frazier K.S., Obert L.A. (2018). Drug-induced Glomerulonephritis: The Spectre of Biotherapeutic and Antisense Oligonucleotide Immune Activation in the Kidney. Toxicol. Pathol..

[50] Moledina D.G., Perazella M.A. (2017). Drug-Induced Acute Interstitial Nephritis. Clin. J. Am. Soc. Nephrol..

[51] Markowitz G.S., Perazella M.A. (2005). Drug-induced renal failure: A focus on tubulointerstitial disease. Clin. Chim. Acta.

[52] Ding H., Li L.X., Harris P.C., Yang J., Li X. (2021). Extracellular vesicles and exosomes generated from cystic renal epithelial cells promote cyst growth in autosomal dominant polycystic kidney disease. Nat. Commun..

[53] Kramann R., Tanaka M., Humphreys B.D. (2014). Fluorescence microangiography for quantitative assessment of peritubular capillary changes after AKI in mice. J. Am. Soc. Nephrol..

[54] Dunn K.W., Sutton T.A., Sandoval R.M. (2018). Live-Animal Imaging of Renal Function by Multiphoton Microscopy. Curr. Protoc. Cytom..

[55] Verma S.K., Molitoris B.A. (2015). Renal endothelial injury and microvascular dysfunction in acute kidney injury. Semin. Nephrol..

[56] Dimke H., Sparks M.A., Thomson B.R., Frische S., Coffman T.M., Quaggin S.E. (2015). Tubulovascular cross-talk by vascular endothelial growth factor a maintains peritubular microvasculature in kidney. J. Am. Soc. Nephrol..

[57] Lameire N. (2014). Nephrotoxicity of recent anti-cancer agents. Clin. Kidney J..

[58] Al-Nouri Z.L., Reese J.A., Terrell D.R., Vesely S.K., George J.N. (2015). Drug-induced thrombotic microangiopathy: A systematic review of published reports. Blood.

[59] Wang P., Sun Y., Shi X., Shen H., Ning H., Liu H. (2021). 3D printing of tissue engineering scaffolds: A focus on vascular regeneration. Biodes Manuf..

[60] Rahman M., Shad F., Smith M.C. (2012). Acute kidney injury: A guide to diagnosis and management. Am. Fam. Physician.

[61] Kellum J.A., Lameire N., Group K.A.G.W. (2013). Diagnosis, evaluation, and management of acute kidney injury: A KDIGO summary (Part 1). Crit. Care.

[62] Port F.K., Eknoyan G. (2004). The Dialysis Outcomes and Practice Patterns Study (DOPPS) and the Kidney Disease Outcomes Quality Initiative (K/DOQI): A cooperative initiative to improve outcomes for hemodialysis patients worldwide. Am. J. Kidney Dis..

[63] Murugan R., Kellum J.A. (2011). Acute kidney injury: What’s the prognosis?. Nat. Rev. Nephrol..

[64] Bajaj P., Chowdhury S.K., Yucha R., Kelly E.J., Xiao G. (2018). Emerging Kidney Models to Investigate Metabolism, Transport, and Toxicity of Drugs and Xenobiotics. Drug Metab. Dispos..

[65] Brown C.D., Sayer R., Windass A.S., Haslam I.S., De Broe M.E., D’Haese P.C., Verhulst A. (2008). Characterisation of human tubular cell monolayers as a model of proximal tubular xenobiotic handling. Toxicol. Appl. Pharmacol..

[66] Tasnim F., Zink D. (2012). Cross talk between primary human renal tubular cells and endothelial cells in cocultures. Am. J. Physiol. Renal. Physiol..

[67] Astashkina A.I., Mann B.K., Prestwich G.D., Grainger D.W. (2012). Comparing predictive drug nephrotoxicity biomarkers in kidney 3-D primary organoid culture and immortalized cell lines. Biomaterials.

[68] Barre-Sinoussi F., Montagutelli X. (2015). Animal models are essential to biological research: Issues and perspectives. Future Sci. OA.

[69] Neves F., Abrantes J., Almeida T., de Matos A.L., Costa P.P., Esteves P.J. (2015). Genetic characterization of interleukins (IL-1alpha, IL-1beta, IL-2, IL-4, IL-8, IL-10, IL-12A, IL-12B, IL-15 and IL-18) with relevant biological roles in lagomorphs. Innate Immun..

[70] Soo J.Y., Jansen J., Masereeuw R., Little M.H. (2018). Advances in predictive in vitro models of drug-induced nephrotoxicity. Nat. Rev. Nephrol..

[71] Justice B.A., Badr N.A., Felder R.A. (2009). 3D cell culture opens new dimensions in cell-based assays. Drug Discov. Today.

[72] Langhans S.A. (2018). Three-Dimensional in Vitro Cell Culture Models in Drug Discovery and Drug Repositioning. Front. Pharmacol..

[73] Riss T., Trask O.J. (2021). Factors to consider when interrogating 3D culture models with plate readers or automated microscopes. In Vitro Cell. Dev. Biol. Anim..

[74] Roelants C., Pillet C., Franquet Q., Sarrazin C., Peilleron N., Giacosa S., Guyon L., Fontanell A., Fiard G., Long J.A. (2020). Ex-Vivo Treatment of Tumor Tissue Slices as a Predictive Preclinical Method to Evaluate Targeted Therapies for Patients with Renal Carcinoma. Cancers.

[75] Kashaninejad N., Chan W.K., Nguyen N.T. (2012). Eccentricity effect of micropatterned surface on contact angle. Langmuir.

[76] Kashaninejad N., Nikmaneshi M.R., Moghadas H., Kiyoumarsi Oskouei A., Rismanian M., Barisam M., Saidi M.S., Firoozabadi B. (2016). Organ-Tumor-on-a-Chip for Chemosensitivity Assay: A Critical Review. Micromachines.

[77] Esch E.W., Bahinski A., Huh D. (2015). Organs-on-chips at the frontiers of drug discovery. Nat. Rev. Drug Discov..

[78] Bhise N.S., Ribas J., Manoharan V., Zhang Y.S., Polini A., Massa S., Dokmeci M.R., Khademhosseini A. (2014). Organ-on-a-chip platforms for studying drug delivery systems. J. Control Release.

[79] Polini A., Prodanov L., Bhise N.S., Manoharan V., Dokmeci M.R., Khademhosseini A. (2014). Organs-on-a-chip: A new tool for drug discovery. Expert Opin. Drug Discov..

[80] Jang K.J., Suh K.Y. (2010). A multi-layer microfluidic device for efficient culture and analysis of renal tubular cells. Lab Chip..

[81] Jang K.J., Mehr A.P., Hamilton G.A., McPartlin L.A., Chung S., Suh K.Y., Ingber D.E. (2013). Human kidney proximal tubule-on-a-chip for drug transport and nephrotoxicity assessment. Integr. Biol..

[82] Yoo T.H., Fornoni A. (2015). Nonimmunologic targets of immunosuppressive agents in podocytes. Kidney Res. Clin. Pract..

[83] Friedrich C., Endlich N., Kriz W., Endlich K. (2006). Podocytes are sensitive to fluid shear stress in vitro. Am. J. Physiol. Renal. Physiol..

[84] Mashanov G.I., Nenasheva T.A., Mashanova T., Maclachlan C., Birdsall N.J.M., Molloy J.E. (2021). A method for imaging single molecules at the plasma membrane of live cells within tissue slices. J. Gen. Physiol..

[85] Kirschnick N., Drees D., Redder E., Erapaneedi R., Pereira da Graca A., Schafers M., Jiang X., Kiefer F. (2021). Rapid methods for the evaluation of fluorescent reporters in tissue clearing and the segmentation of large vascular structures. iScience.

[86] Freedman B.S., Brooks C.R., Lam A.Q., Fu H., Morizane R., Agrawal V., Saad A.F., Li M.K., Hughes M.R., Werff R.V. (2015). Modelling kidney disease with CRISPR-mutant kidney organoids derived from human pluripotent epiblast spheroids. Nat. Commun..

[87] Wilmer M.J., Ng C.P., Lanz H.L., Vulto P., Suter-Dick L., Masereeuw R. (2016). Kidney-on-a-Chip Technology for Drug-Induced Nephrotoxicity Screening. Trends Biotechnol..

[88] Taguchi A., Kaku Y., Ohmori T., Sharmin S., Ogawa M., Sasaki H., Nishinakamura R. (2014). Redefining the in vivo origin of metanephric nephron progenitors enables generation of complex kidney structures from pluripotent stem cells. Cell Stem Cell.

[89] Schutgens F., Rookmaaker M.B., Margaritis T., Rios A., Ammerlaan C., Jansen J., Gijzen L., Vormann M., Vonk A., Viveen M. (2019). Tubuloids derived from human adult kidney and urine for personalized disease modeling. Nat. Biotechnol..

[90] Yoshimura Y., Taguchi A., Tanigawa S., Yatsuda J., Kamba T., Takahashi S., Kurihara H., Mukoyama M., Nishinakamura R. (2019). Manipulation of Nephron-Patterning Signals Enables Selective Induction of Podocytes from Human Pluripotent Stem Cells. J. Am. Soc. Nephrol..

[91] Breiderhoff T., Himmerkus N., Stuiver M., Mutig K., Will C., Meij I.C., Bachmann S., Bleich M., Willnow T.E., Muller D. (2012). Deletion of claudin-10 (Cldn10) in the thick ascending limb impairs paracellular sodium permeability and leads to hypermagnesemia and nephrocalcinosis. Proc. Natl. Acad. Sci. USA.

[92] Dimke H., Desai P., Borovac J., Lau A., Pan W., Alexander R.T. (2013). Activation of the Ca^2+^-sensing receptor increases renal claudin-14 expression and urinary Ca^2+^ excretion. Am. J. Physiol. Renal. Physiol..

[93] Olofsson B., Korpelainen E., Pepper M.S., Mandriota S.J., Aase K., Kumar V., Gunji Y., Jeltsch M.M., Shibuya M., Alitalo K. (1998). Vascular endothelial growth factor B (VEGF-B) binds to VEGF receptor-1 and regulates plasminogen activator activity in endothelial cells. Proc. Natl. Acad. Sci. USA.

[94] Low J.H., Li P., Chew E.G.Y., Zhou B., Suzuki K., Zhang T., Lian M.M., Liu M., Aizawa E., Rodriguez Esteban C. (2019). Generation of Human PSC-Derived Kidney Organoids with Patterned Nephron Segments and a De Novo Vascular Network. Cell Stem Cell..

[95] Hale L.J., Howden S.E., Phipson B., Lonsdale A., Er P.X., Ghobrial I., Hosawi S., Wilson S., Lawlor K.T., Khan S. (2018). 3D organoid-derived human glomeruli for personalised podocyte disease modelling and drug screening. Nat. Commun..

[96] Bulow R.D., Boor P. (2019). Extracellular Matrix in Kidney Fibrosis: More Than Just a Scaffold. J. Histochem. Cytochem..

[97] Finesilver G., Bailly J., Kahana M., Mitrani E. (2014). Kidney derived micro-scaffolds enable HK-2 cells to develop more in-vivo like properties. Exp. Cell Res..

[98] Bonandrini B., Figliuzzi M., Papadimou E., Morigi M., Perico N., Casiraghi F., Dipl C., Sangalli F., Conti S., Benigni A. (2014). Recellularization of well-preserved acellular kidney scaffold using embryonic stem cells. Tissue Eng. Part A.

[99] Batchelder C.A., Martinez M.L., Tarantal A.F. (2015). Natural Scaffolds for Renal Differentiation of Human Embryonic Stem Cells for Kidney Tissue Engineering. PLoS ONE.

[100] Uzarski J.S., Su J., Xie Y., Zhang Z.J., Ward H.H., Wandinger-Ness A., Miller W.M., Wertheim J.A. (2015). Epithelial Cell Repopulation and Preparation of Rodent Extracellular Matrix Scaffolds for Renal Tissue Development. J. Vis. Exp..

[101] Kharkar P.M., Kiick K.L., Kloxin A.M. (2013). Designing degradable hydrogels for orthogonal control of cell microenvironments. Chem. Soc. Rev..

[102] Van Vlierberghe S., Dubruel P., Schacht E. (2011). Biopolymer-based hydrogels as scaffolds for tissue engineering applications: A review. Biomacromolecules.

[103] Lee K., Silva E.A., Mooney D.J. (2011). Growth factor delivery-based tissue engineering: General approaches and a review of recent developments. J. R. Soc. Interface.

[104] Nichol J.W., Koshy S.T., Bae H., Hwang C.M., Yamanlar S., Khademhosseini A. (2010). Cell-laden microengineered gelatin methacrylate hydrogels. Biomaterials.

[105] Jansen K., Schuurmans C.C.L., Jansen J., Masereeuw R., Vermonden T. (2017). Hydrogel-Based Cell Therapies for Kidney Regeneration: Current Trends in Biofabrication and In Vivo Repair. Curr. Pharm. Des..

[106] D’Costa K., Kosic M., Lam A., Moradipour A., Zhao Y., Radisic M. (2020). Biomaterials and Culture Systems for Development of Organoid and Organ-on-a-Chip Models. Ann. Biomed. Eng..

[107] Cai Z., Xin J., Pollock D.M., Pollock J.S. (2000). Shear stress-mediated NO production in inner medullary collecting duct cells. Am. J. Physiol. Renal. Physiol..

[108] Raghavan V., Weisz O.A. (2016). Discerning the role of mechanosensors in regulating proximal tubule function. Am. J. Physiol. Renal. Physiol..

[109] Ong L.J.Y., Zhu L., Tan G.J.S., Toh Y.C. (2020). Quantitative Image-Based Cell Viability (QuantICV) Assay for Microfluidic 3D Tissue Culture Applications. Micromachines.

[110] Vriend J., Nieskens T.T.G., Vormann M.K., van den Berge B.T., van den Heuvel A., Russel F.G.M., Suter-Dick L., Lanz H.L., Vulto P., Masereeuw R. (2018). Screening of Drug-Transporter Interactions in a 3D Microfluidic Renal Proximal Tubule on a Chip. AAPS J..

[111] Theobald J., Ghanem A., Wallisch P., Banaeiyan A.A., Andrade-Navarro M.A., Taskova K., Haltmeier M., Kurtz A., Becker H., Reuter S. (2018). Liver-Kidney-on-Chip To Study Toxicity of Drug Metabolites. ACS Biomater. Sci. Eng..

[112] Duzagac F., Saorin G., Memeo L., Canzonieri V., Rizzolio F. (2021). Microfluidic Organoids-on-a-Chip: Quantum Leap in Cancer Research. Cancers.

[113] Ryan M.J., Johnson G., Kirk J., Fuerstenberg S.M., Zager R.A., Torok-Storb B. (1994). HK-2: An immortalized proximal tubule epithelial cell line from normal adult human kidney. Kidney Int..

[114] Prozialeck W.C., Edwards J.R., Lamar P.C., Smith C.S. (2006). Epithelial barrier characteristics and expression of cell adhesion molecules in proximal tubule-derived cell lines commonly used for in vitro toxicity studies. Toxicol. In Vitro.

[115] Kim Y.K., Nam S.A., Yang C.W. (2018). Applications of kidney organoids derived from human pluripotent stem cells. Korean J. Intern. Med..

[116] Prozialeck W.C., Edwards J.R. (2007). Cell adhesion molecules in chemically-induced renal injury. Pharmacol. Ther..

[117] Nieskens T.T., Peters J.G., Schreurs M.J., Smits N., Woestenenk R., Jansen K., van der Made T.K., Roring M., Hilgendorf C., Wilmer M.J. (2016). A Human Renal Proximal Tubule Cell Line with Stable Organic Anion Transporter 1 and 3 Expression Predictive for Antiviral-Induced Toxicity. AAPS J..

[118] Stray K.M., Bam R.A., Birkus G., Hao J., Lepist E.I., Yant S.R., Ray A.S., Cihlar T. (2013). Evaluation of the effect of cobicistat on the in vitro renal transport and cytotoxicity potential of tenofovir. Antimicrob. Agents Chemother..

[119] Uetake R., Sakurai T., Kamiyoshi A., Ichikawa-Shindo Y., Kawate H., Iesato Y., Yoshizawa T., Koyama T., Yang L., Toriyama Y. (2014). Adrenomedullin-RAMP2 system suppresses ER stress-induced tubule cell death and is involved in kidney protection. PLoS ONE.

[120] Takasato M., Er P.X., Becroft M., Vanslambrouck J.M., Stanley E.G., Elefanty A.G., Little M.H. (2014). Directing human embryonic stem cell differentiation towards a renal lineage generates a self-organizing kidney. Nat. Cell Biol..

[121] Lam A.Q., Freedman B.S., Morizane R., Lerou P.H., Valerius M.T., Bonventre J.V. (2014). Rapid and efficient differentiation of human pluripotent stem cells into intermediate mesoderm that forms tubules expressing kidney proximal tubular markers. J. Am. Soc. Nephrol..

[122] Liu G., Wu R., Yang B., Deng C., Lu X., Walker S.J., Ma P.X., Mou S., Atala A., Zhang Y. (2018). Human Urine-Derived Stem Cell Differentiation to Endothelial Cells with Barrier Function and Nitric Oxide Production. Stem Cells Transl. Med..

[123] Zhang Y., McNeill E., Tian H., Soker S., Andersson K.E., Yoo J.J., Atala A. (2008). Urine derived cells are a potential source for urological tissue reconstruction. J. Urol..

[124] Bharadwaj S., Liu G., Shi Y., Wu R., Yang B., He T., Fan Y., Lu X., Zhou X., Liu H. (2013). Multipotential differentiation of human urine-derived stem cells: Potential for therapeutic applications in urology. Stem Cells.

[125] Han H.J., Sigurdson W.J., Nickerson P.A., Taub M. (2004). Both mitogen activated protein kinase and the mammalian target of rapamycin modulate the development of functional renal proximal tubules in matrigel. J. Cell Sci..

[126] Arakawa H., Washio I., Matsuoka N., Kubo H., Staub A.Y., Nakamichi N., Ishiguro N., Kato Y., Nakanishi T., Tamai I. (2017). Usefulness of kidney slices for functional analysis of apical reabsorptive transporters. Sci. Rep..

[127] McNeil E., Capaldo C.T., Macara I.G. (2006). Zonula occludens-1 function in the assembly of tight junctions in Madin-Darby canine kidney epithelial cells. Mol. Biol. Cell.

[128] Gunness P., Aleksa K., Kosuge K., Ito S., Koren G. (2010). Comparison of the novel HK-2 human renal proximal tubular cell line with the standard LLC-PK1 cell line in studying drug-induced nephrotoxicity. Can. J. Physiol. Pharmacol..

[129] McClane B.A., Chakrabarti G. (2004). New insights into the cytotoxic mechanisms of Clostridium perfringens enterotoxin. Anaerobe.

[130] Iuchi K., Oya K., Hosoya K., Sasaki K., Sakurada Y., Nakano T., Hisatomi H. (2020). Different morphologies of human embryonic kidney 293T cells in various types of culture dishes. Cytotechnology.

[131] Prange J.A., Bieri M., Segerer S., Burger C., Kaech A., Moritz W., Devuyst O. (2016). Human proximal tubule cells form functional microtissues. Pflug. Arch..

[132] Guan L., Fan P., Liu X., Liu R., Liu Y., Bai H. (2021). Migration of Human Renal Tubular Epithelial Cells in Response to Physiological Electric Signals. Front. Cell Dev. Biol..

[133] Secker P.F., Luks L., Schlichenmaier N., Dietrich D.R. (2018). RPTEC/TERT1 cells form highly differentiated tubules when cultured in a 3D matrix. ALTEX.

[134] Jansen J., Fedecostante M., Wilmer M.J., Peters J.G., Kreuser U.M., van den Broek P.H., Mensink R.A., Boltje T.J., Stamatialis D., Wetzels J.F. (2016). Bioengineered kidney tubules efficiently excrete uremic toxins. Sci. Rep..

[135] Fedecostante M., Onciu O.G., Westphal K.G.C., Masereeuw R. (2017). Towards a bioengineered kidney: Recellularization strategies for decellularized native kidney scaffolds. Int. J. Artif. Organs.

[136] O’Brien L.E., Zegers M.M., Mostov K.E. (2002). Opinion: Building epithelial architecture: Insights from three-dimensional culture models. Nat. Rev. Mol. Cell Biol..

[137] Imai M., Furusawa K., Mizutani T., Kawabata K., Haga H. (2015). Three-dimensional morphogenesis of MDCK cells induced by cellular contractile forces on a viscous substrate. Sci. Rep..

[138] Su G., Zhao Y., Wei J., Han J., Chen L., Xiao Z., Chen B., Dai J. (2013). The effect of forced growth of cells into 3D spheres using low attachment surfaces on the acquisition of stemness properties. Biomaterials.

[139] Hueso M., Navarro E., Sandoval D., Cruzado J.M. (2019). Progress in the Development and Challenges for the Use of Artificial Kidneys and Wearable Dialysis Devices. Kidney Dis..

[140] Terashima M., Fujita Y., Sugano K., Asano M., Kagiwada N., Sheng Y., Nakamura S., Hasegawa A., Kakuta T., Saito A. (2001). Evaluation of water and electrolyte transport of tubular epithelial cells under osmotic and hydraulic pressure for development of bioartificial tubules. Artif. Organs.

[141] Ueda H., Watanabe J., Konno T., Takai M., Saito A., Ishihara K. (2006). Asymmetrically functional surface properties on biocompatible phospholipid polymer membrane for bioartificial kidney. J. Biomed. Mater. Res. A.

[142] Nair A.L., Mesch L., Schulz I., Becker H., Raible J., Kiessling H., Werner S., Rothbauer U., Schmees C., Busche M. (2021). Parallelizable Microfluidic Platform to Model and Assess In Vitro Cellular Barriers: Technology and Application to Study the Interaction of 3D Tumor Spheroids with Cellular Barriers. Biosensors.

[143] Chuva de Sousa Lopes S.M. (2019). Accelerating maturation of kidney organoids. Nat. Mater..

[144] Wan Q., Xiong G., Liu G., Shupe T.D., Wei G., Zhang D., Liang D., Lu X., Atala A., Zhang Y. (2018). Urothelium with barrier function differentiated from human urine-derived stem cells for potential use in urinary tract reconstruction. Stem Cell Res. Ther..

[145] O’Brien L.L., Combes A.N., Short K.M., Lindstrom N.O., Whitney P.H., Cullen-McEwen L.A., Ju A., Abdelhalim A., Michos O., Bertram J.F. (2018). Wnt11 directs nephron progenitor polarity and motile behavior ultimately determining nephron endowment. Elife.

[146] Morizane R., Bonventre J.V. (2017). Kidney Organoids: A Translational Journey. Trends Mol. Med..

[147] Taguchi A., Nishinakamura R. (2017). Higher-Order Kidney Organogenesis from Pluripotent Stem Cells. Cell Stem Cell.

[148] Ryan A.R., England A.R., Chaney C.P., Cowdin M.A., Hiltabidle M., Daniel E., Gupta A.K., Oxburgh L., Carroll T.J., Cleaver O. (2021). Vascular deficiencies in renal organoids and ex vivo kidney organogenesis. Dev. Biol..

[149] Hariharan K., Reinke P., Kurtz A. (2019). Generating Multiple Kidney Progenitors and Cell Types from Human Pluripotent Stem Cells. Methods Mol. Biol..

[150] Magella B., Adam M., Potter A.S., Venkatasubramanian M., Chetal K., Hay S.B., Salomonis N., Potter S.S. (2018). Cross-platform single cell analysis of kidney development shows stromal cells express Gdnf. Dev. Biol..

[151] Bagherie-Lachidan M., Reginensi A., Pan Q., Zaveri H.P., Scott D.A., Blencowe B.J., Helmbacher F., McNeill H. (2015). Stromal Fat4 acts non-autonomously with Dchs1/2 to restrict the nephron progenitor pool. Development.

[152] Mao Y., Francis-West P., Irvine K.D. (2015). Fat4/Dchs1 signaling between stromal and cap mesenchyme cells influences nephrogenesis and ureteric bud branching. Development.

[153] Humphreys B.D., Lin S.L., Kobayashi A., Hudson T.E., Nowlin B.T., Bonventre J.V., Valerius M.T., McMahon A.P., Duffield J.S. (2010). Fate tracing reveals the pericyte and not epithelial origin of myofibroblasts in kidney fibrosis. Am. J. Pathol..

[154] Kobayashi A., Mugford J.W., Krautzberger A.M., Naiman N., Liao J., McMahon A.P. (2014). Identification of a multipotent self-renewing stromal progenitor population during mammalian kidney organogenesis. Stem Cell Rep..

[155] Lemos D.R., McMurdo M., Karaca G., Wilflingseder J., Leaf I.A., Gupta N., Miyoshi T., Susa K., Johnson B.G., Soliman K. (2018). Interleukin-1beta Activates a MYC-Dependent Metabolic Switch in Kidney Stromal Cells Necessary for Progressive Tubulointerstitial Fibrosis. J. Am. Soc. Nephrol..

